# Dextran Fractional Clearance Studies in Acute Dengue Infection

**DOI:** 10.1371/journal.pntd.0001282

**Published:** 2011-08-23

**Authors:** Julie Nguyen-Pouplin, Thomas Pouplin, Toi Pham Van, Trung Dinh The, Dung Nguyen Thi, Jeremy Farrar, Hien Tran Tinh, Bridget Wills

**Affiliations:** 1 Wellcome Trust Major Overseas Programme, Oxford University Clinical Research Unit, Ho Chi Minh City, Viet Nam; 2 Hospital for Tropical Diseases, Ho Chi Minh City, Viet Nam; 3 Centre for Clinical Vaccinology and Tropical Medicine, Oxford University, Oxford, United Kingdom; 4 University of Medicine and Pharmacy of Ho Chi Minh City, Ho Chi Minh City, Viet Nam; University of California, Berkeley, United States of America

## Abstract

**Background:**

Although increased capillary permeability is the major clinical feature associated with severe dengue infections the mechanisms underlying this phenomenon remain unclear. Dextran clearance methodology has been used to investigate the molecular sieving properties of the microvasculature in clinical situations associated with altered permeability, including during pregnancy and in various renal disorders. In order to better understand the characteristics of the vascular leak associated with dengue we undertook formal dextran clearance studies in Vietnamese dengue patients and healthy volunteers.

**Methodology/Principal Findings:**

We carried out serial clearance studies in 15 young adult males with acute dengue and evidence of vascular leakage a) during the phase of maximal leakage and b) one and three months later, as well as in 16 healthy control subjects. Interestingly we found no difference in the clearance profiles of neutral dextran solutions among the dengue patients at any time-point or in comparison to the healthy volunteers.

**Conclusions/Significance:**

The surface glycocalyx layer, a fibre-matrix of proteoglycans, glycosaminoglycans, and plasma proteins, forms a complex with the underlying endothelial cells to regulate plasma volume within circumscribed limits. It is likely that during dengue infections loss of plasma proteins from this layer alters the permeability characteristics of the complex; physical and/or electrostatic interactions between the dextran molecules and the glycocalyx structure may temporarily restore normal function, rendering the technique unsuitable for assessing permeability in these patients. The implications for resuscitation of patients with dengue shock syndrome (DSS) are potentially important. It is possible that continuous low-dose infusions of dextran may help to stabilize the permeability barrier in patients with profound or refractory shock, reducing the need for repeated boluses, limiting the total colloid volume required. Formal clinical studies should help to assess this strategy as an alternative to conventional fluid resuscitation for severe DSS.

## Author Summary

Dengue is a potentially serious common viral infection with no specific treatment. Plasma leakage from small blood vessels is the major severe problem, but we do not understand how this occurs. Techniques using controlled infusions of carbohydrate solutions, combined with careful measurement of the rate that the different-sized molecules clear from the circulation, have been successfully used to investigate leakage in other situations. We performed carbohydrate clearance studies in 15 Vietnamese adult males with dengue and plasma leakage, comparing results obtained during the acute illness with recovery values, and results from a group of healthy volunteers. However, we found no differences between any of the clearance profiles measured. One possible explanation may be that the carbohydrate molecules interact with blood vessels, temporarily restoring their normal barrier function. Although this means that the technique is unsuitable for investigating leakage in dengue patients, the implications for management of patients with severe leakage resulting in shock are potentially important. Patients with profound shock are usually managed with intermittent large boluses of carbohydrate or similar solutions, sometimes causing severe side-effects; however if continuous low-dose infusions actually stabilized the permeability barrier, this might reduce the need for repeated boluses, thereby minimizing these adverse effects.

## Introduction

Dengue infection is increasingly being recognised as a major burden on global health [1]. Infection with any of the four viral serotypes may result in asymptomatic infection, or cause a variety of disease manifestations ranging from non-specific fever to a severe syndrome characterised by increased vascular permeability, deranged haemostasis and thrombocytopenia [2]. Considerable volumes of plasma can be lost from the intravascular compartment resulting in potentially fatal dengue shock syndrome. Despite the fact that vascular leakage is the pathognomonic feature of severe dengue, little is known of the mechanisms underlying the change in permeability. The prevailing view is that dengue infection triggers an immunopathogenic cascade that alters microvascular structure or function in some as yet undefined way, resulting in a transient, spontaneously-reversible increase in permeability [3].

Plasma volume is normally regulated within tightly circumscribed limits by complex homeostatic mechanisms, with plasma and interstitial fluid existing in dynamic equilibrium separated by the semi-permeable capillary wall [4], [5]. The surface glycocalyx layer, a highly anionic fibre-matrix of proteoglycans, glycosaminoglycans, and plasma proteins anchored in the plasma membrane of endothelial cells, is considered to be the primary barrier, functioning as a molecular sieve to selectively restrict molecules within the plasma and limit access to the endothelial cell layer which forms the secondary barrier. In general, small molecules are freely filtered, permeability decreases as molecular size increases, and large or negatively charged molecules are relatively protected within the circulation.

Although the basic mechanisms governing filtration at the glomerulus are intrinsically similar to those operating in the rest of the microvascular circulation, additional highly specialised mechanisms serve to protect the intravascular albumin pool in the face of a glomerular filtration rate of the order of 180 litres/day in adults, resulting in production of virtually protein free urine in normal circumstances [5], [6]. Increases in glomerular protein permeability demonstrated in patients without renal disease are likely to indicate substantial increases in systemic vascular permeability. Recent research in various diseases with altered capillary permeability such as diabetes and coronary atherosclerosis indicates a range of pathological effects on the surface glycocalyx layer of the systemic microvasculature, with early microalbuminuria thought to reflect these systemic changes rather than being directly of renal origin [7]–[10].

Knowledge of the sieving properties of capillary beds can be obtained by measuring the fractional clearance of test macromolecules [11], [12]. The clearance of a molecule is equal to the excretion in urine per unit time divided by the concentration in plasma, provided no modification occurs in the kidneys. Relating this clearance to that of a freely filtered reference marker that is neither secreted nor absorbed adjusts for the glomerular filtration rate. Since many endogenous proteins are secreted and/or absorbed in the renal tubules, fractional clearance measurements of non-reabsorbable synthetic polymers such as dextrans are preferred.

Both hypoalbuminaemia and proteinuria are seen during dengue infections, without evidence of renal impairment. Marked increases in fractional clearances of several endogenous proteins, notably albumin, have been documented during the critical phase for leakage [13]. In order to better understand the characteristics of the vascular leak associated with dengue, we undertook formal dextran clearance studies in a group of Vietnamese patients with dengue and a similar group of healthy volunteers as controls.

## Methods

### Clinical Methods

Serial dextran clearance studies were carried out on adult male patients at the Hospital for Tropical Diseases (HTD) of Ho Chi Minh City, all with dengue and evidence of vascular leakage, a) during the phase of maximal leakage and b) one and three months later. Similar clearance studies were performed on healthy male volunteers with no history of febrile illness for 3 months. All subjects gave written informed consent, and approval was obtained from HTD and the Oxford Tropical Research Ethics Committee. All studies took place in a dedicated pharmacology research room on a high-dependency ward supervised by an experienced clinician.

Following established methodology, a 50 ml bolus of 10% Dextran 40 in normal saline (Rheopolyglukin, Kraspharma, Russia) plus 5% inulin (Inutest® 25%, Fresenius Kabi, Austria) was administered over 10 minutes to supine resting subjects, followed by a maintenance infusion of the same solution at 1 ml/min for 2 hours [12]. Inulin is commonly used as the standard for measuring ultrafiltration of small solutes. Blood samples (2 mL) were collected from the contralateral arm at baseline, and again after 70 and 130 minutes, to coincide with the mid-point of timed urine collections performed between 60–80 and 120–140 minutes immediately after the subject had voided the bladder. Subjects were encouraged to drink plenty of water and vital signs were recorded before and every 30 minutes during the test. The exact timings of fluid administration and sample collection were carefully recorded. Samples were immediately separated and stored at −20°C until analysed subsequently in batches.

### Sample analysis

All samples from one subject were analysed on the same day by high performance liquid chromatography (HPLC) using a LaChrom Elite system (Merck, Germany) including one auto-sampler with cooling unit (L-2200), two high throughput analysis pumps (model L-2130), a column oven (model L-2350) and a Diode Array Detector (model L-2455). All plasma and urine samples were first deproteinized with 20% trichloroacetic acid.

#### Dextrans

Polydisperse dextrans were separated into narrow fractions according to their molecular size by HPLC, with two columns Ultrahydrogel 500 and 250 in series protected by a guard column (Waters Corp., USA) [11], infusing a 0.02 M phosphate buffer (pH 7). Chromatography was carried out at 35°C, with a flow rate at 0.5 mL/min and UV detection at 625 nm. Multiple eluting fractions (250 µL) were collected between 25–45 minutes, and the dextran concentrations were estimated by colorimetry using anthrone assays [14]. The columns were calibrated using 6 standard dextrans ranging from 5–500 kD (Pharmacosmos, Denmark), and the void volume (V_0_) was determined with Blue Dextran (Sigma, USA).

#### Inulin

After pre-treatment with perchloric acid, inulin concentrations were measured by HPLC using a LichroCart RP-18 column and its corresponding guard column (Merck, Germany) and a 0.01 M phosphate buffer in 4% acetonitrile [15]. Chromatography was carried out on 20 µL of each sample over 8 minutes at 1 mL/min, giving an inulin peak retention time at 5.21 minutes (λ = 285 nm). Precision and accuracy were acceptable with a coefficient of variability <15%.

### Fractional clearance calculations

Fractional clearances of dextran were calculated as follows: θ_Dex_ = (U/P)_Dex_/(U/P)_Inu_, which refer to the urine-to-midpoint plasma concentration ratios of dextran and inulin respectively. The fractional volume was determined as K_av_ = (V_e_−V_0_)/(V_t_−V_0_), where V_e_ is the elution volume of each dextran in individual fractions and V_t_ is the total volume of the column, estimated with glucose. The molecular radius was calculated by R_st_ = 0.33×M^0.463^, M being the molecular weight at the peak elution position of each standard dextran. R_st_ of individual fractions was estimated by the relationship between K_av_ and R_st_ of the standard dextran solutions.

## Results

Between November 2008 and February 2009 15 male patients, median (range) 25 (18–30) years, were recruited to the study. All had clinically suspected dengue with evidence of vascular leakage (progressively rising haematocrit (11/15) and/or pleural effusion or ascites on ultrasound (5/15) and/or hypoalbuminaemia (9/15)) at the time of study, but were well-compensated cardiovascularly. One patient was diagnosed as having vascular leakage on the basis of development of significant hypoalbuminaemia alone, with the plasma albumin dropping from 47 g/L to 39.5 g/L on day 4 of illness. This patient received maintenance parenteral fluid therapy, likely masking the rise in haematocrit usually seen in association with such a reduction in plasma albumin. All initial studies took place between days 4–6 of illness (the first day of illness was defined as the day of fever onset), with 13/15 on day 5. No patient developed shock and all recovered fully with conventional symptomatic care. Throughout the course of the illness 11/15 patients experienced either skin or minor mucosal bleeding and the median (range) platelet nadir was 25,000 (8,000–111,000) cells/µl. All patients were subsequently confirmed to have dengue using standard serological/virological methods (5 patients sero-converted, while in 10 a virus was identified on RT-PCR, 5 DENV_1, 3 DENV_2 and 2 DENV_3) [16]. Sixteen healthy male students, median (range) 24 (23–28) years, acted as controls. Clearance studies were generally well tolerated although one patient developed a minor febrile reaction shortly after the dextran infusion.

**Figure 1:**
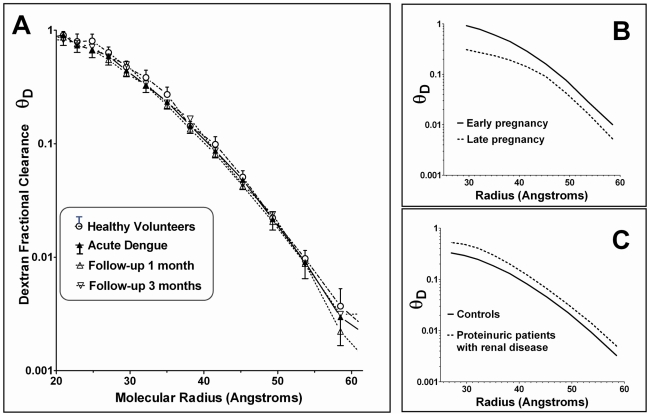
Dextran fractional clearances. **Panel A:** Mean and standard deviation dextran fractional clearances (θ_D_) for dengue patients during the acute phase (n = 15), and at 1 month (n = 15) and 3 months (n = 14) following recovery, compared with healthy volunteers (n = 16). For reasons of clarity the standard deviation markers are only presented for the acute dengue results, and for the healthy volunteers. **Panels B and C:** For comparison, these panels depict schematic examples of typical patterns seen when clearances are reduced, for example during pregnancy, or increased, for example in patients with renal disorders.

Figure 1 depicts the dextran fractional clearance curves for the acute and convalescent studies performed in the dengue patients, together with results for the control subjects. All curves represent the results from the first timed collection. No detectable differences were apparent between any of the curves. There were no differences in inulin clearances between the groups during the acute illness or between acute illness measurements and follow-up measurements within any of the groups, consistent with the persistently normal renal function indices observed in all patients throughout the duration of the study.

## Discussion

Fractional clearance methodology is well established for investigating renal disorders and has provided useful insights into the pathogenesis of the systemic leak associated with meningococcal septicaemia and dengue, in both of which considerable increases in clearances of endogenous proteins were observed consistent with the severity of leakage [13], [17]. However, in these formal dextran clearance studies in dengue patients we found no difference in the clearance profiles of polydisperse neutral dextran solutions either a) during the period of maximal leakage compared to their own follow-up profiles, or b) compared to the profiles observed in a similar group of healthy volunteers. Although all the patients had clear evidence of leakage, none developed DSS and they were thus comparatively less severe than the patient group we previously studied, in whom marked increases in protein clearances were demonstrated in association with DSS. Unfortunately nevertheless we did not measure clearances of endogenous proteins prior to the dextran clearance studies so we cannot be sure that they did have increased protein losses at baseline; however ongoing studies in our unit indicate that all patients with dengue have some degree of microalbuminuria and that most patients with demonstrable vascular leakage have proteinuria on dipstick testing.

One possible explanation for this unexpected finding of normal dextran clearances during the period of vascular leakage is that a physical and/or electrostatic interaction occurs between the dextran molecules and the surface glycocalyx/endothelial cell complex, temporarily improving its permeability characteristics during the infusion and rendering the technique unsuitable for assessing permeability in such patients. Both structural and functional characteristics of the glycocalyx are known to depend in part on integration of endogenous plasma proteins, especially albumin, within the layer [4], [6]. Although the pathogenesis of dengue associated vascular leakage remains unknown, significant losses of albumin and other proteins from the circulating plasma occur, and some washout of proteins from the glycocalyx layer must follow, compromising the function of the layer. However, replacement of lost proteins by synthetic colloid molecules may temporarily restore the permeability characteristics of the barrier. From animal studies incorporation of small dextran molecules into the glycocalyx layer is known to occur almost immediately, while larger molecules usually remain restricted within the circulating plasma for several hours [18]. In situations where the protein content of the glycocalyx is markedly reduced, it is possible that the rate and range of colloid molecules rapidly entering the layer increases. Colloid infusions have been shown to restore permeability in a pig heart model of ischaemia/reperfusion injuries [19], [20], and clinical experience using neutral dextran solutions for resuscitating patients with DSS indicates that the volume effect of a given bolus considerably exceeds the actual volume infused, supporting the idea that colloid molecules transiently restore the permeability barrier thereby reducing the leakage of proteins temporarily [21].

In recent years there has been increasing interest in investigating the mechanisms and regulation of glycocalyx synthesis and turnover, in the hope that such knowledge might lead to the development of novel therapeutic strategies to reduce pathologically increased permeability [5]. Thus, heparin injections are known to mitigate the severe protein losing enteropathy that develops in some children following complex cardiac surgery due to loss of heparan sulfate proteoglycans from the intestinal epithelial glycocalyx [22], [23]. Use of heparin is limited by adverse effects, but alternative strategies to restore gut epithelial glycocalyx function using synthetic heparin-like compounds are being actively pursued [24]. Similarly, alterations to the glycocalyx layer in the coronary microcirculation are thought to contribute to myocardial ischaemia and the subsequent reperfusion injuries that result in localised vascular leakage; prevention of glycocalyx loss and/or restoration of damaged glycocalyx are being investigated as potential interventions to reduce myocardial damage in these circumstances [20], [25], [26].

The implications of these findings for resuscitation of patients with DSS are potentially important. Prompt restoration of circulating plasma volume is the cornerstone of therapy, and WHO management guidelines recommend initial resuscitation with crystalloid solutions followed by boluses of colloids for patients with recurrent or refractory shock, aiming to achieve cardiovascular stability with the minimum volume necessary to maintain vital organ functions until normal permeability is restored [2], [17]. However, patients with severe leakage are intrinsically difficult to manage and often require multiple boluses of colloid during this period, putting them at significant risk of respiratory distress due to fluid overload, and of haemorrhagic complications due to colloid induced haemostatic dysfunction compounding the intrinsic coagulopathy and thrombocytopenia induced by dengue infection. These results indicate that an alternative resuscitation strategy may be beneficial in some circumstances – in severe cases continuous low-dose infusions of dextran may help to stabilise the permeability barrier, reducing the need for repeated boluses and limiting the total volume of colloid infused, thereby minimising adverse effects on coagulation and respiratory function. Further work is needed to confirm these findings in patients with demonstrably increased fractional protein clearances and to investigate whether the effect is similar with other colloid solutions. If the findings are confirmed, formal randomised and blinded intervention studies should help to address the question of whether continuous low dose colloid infusions improve outcome in severe DSS.
